# Religious Affiliation, Internalized Homonegativity and Depressive Symptoms: Unveiling Mental Health Inequalities among Brazilian Gay Men

**DOI:** 10.3390/ijerph21091167

**Published:** 2024-09-02

**Authors:** Felipe Alckmin-Carvalho, António Oliveira, Patricia Silva, Madalena Cruz, Lúcia Nichiata, Henrique Pereira

**Affiliations:** 1 Department of Psychology and Education, Faculty of Social and Human Sciences, University of Beira Interior, Pólo IV, 6200-209 Covilhã, Portugal; antonio.oliveira@ubi.pt (A.O.); pg.silva@ubi.pt (P.S.); madalena.cruz@ubi.pt (M.C.); 2 School of Nursing, University of São Paulo, São Paulo 01239-020, Brazil; izumi@usp.br; 3 Research Center in Sports Sciences, Health Sciences and Human Development (CIDESD), 5001-801 Vila Real, Portugal

**Keywords:** male homosexuality, homonegativity, stigma, depression, religion, religiosity, sexual stigma, LGBTQIA+, mental health, cross-sectional study

## Abstract

Introduction: Different religious narratives associate same-sex sexuality, in its various manifestations, with moral deviation or sin. Gay men who are socialized in more religious communities appear to experience and internalize greater levels of homonegativity, as well as to present greater indicators of depressive symptoms. The purpose of this study was to evaluate indicators of perceived homonegativity in the community and internalized, and signs/symptoms of depression reported by Brazilian gay men with a nominal religion and compare them to those reported by Atheists or Agnostics. Method: Our sample comprised 194 Brazilian gay men, distributed into three groups: Christians (Protestants and Catholics, n = 71; 36.6%); Spiritualists (Kardecists or religions of African origin, n = 52; 26.8%) and Atheists or Agnostics (n = 71; 36.6%). The following measurement instruments were used: sociodemographic questionnaire, Internalized Homophobia Scale and Beck Depression Scale. Results: High mean scores of depression were verified in all groups, and 60% of the sample presented some level of depression. There was a higher level of self-reported homonegativity among Christians and Spiritualists compared to that reported by Atheists or Agnostics, with the differences between the groups being significant. The regression analysis indicated a significant effect of religion on homonegativity, but not on depression. Conclusion: Our results suggest that gay men’s chronic exposure to non-affirming religious affiliation contexts may harm the construction of a positive gay identity and should be taken into consideration when addressing mental health inequalities of sexual minorities.

## 1. Introduction

Homonegativity is characterized by negative reactions and prejudiced beliefs related to homosexuality, which can be expressed in social isolation, physical and verbal violence, or even in less obvious forms of stigma [1,2]. Therefore, the term “homonegativity” offers a perspective that encompasses, in addition to overt forms of discrimination and violence, more subtle forms of prejudice, discrimination and symbolic violence, considered microaggressions [2]. This phenomenon and its various manifestations remain frequent in Latin American countries, especially in Brazil [3,4,5] and are associated with various negative mental health outcomes, impairments in adaptive functioning, reduction in self-reported quality of life, higher frequency of suicidal ideation, suicide attempts, and death by suicide [6,7,8,9,10,11,12]. In addition, there is no shortage of evidence of the mental health disparities associated with non-heterosexual sexual orientation, and their pervasive effects throughout human development, which also negatively affect the level of life satisfaction [13], perception of meaning in life [14], occupational health [15] and satisfaction with affective and sexual relationships [16], with these effects being modulated by the level of structural stigma in the community against sexual minorities.

Although progress has been made on the LGBTQIA+ rights agenda in recent decades in Brazil, such as the criminalization of acts of violence directed at the LGBTQIA+ population by the Brazilian Supreme Court [17], the right to same-sex marriage [18] and the prohibition by the federal councils of medicine and psychology of therapies to reverse homosexual orientation [19], homonegativity remains widespread among the Brazilian community [20]. For example, a recent study [21], which assessed perceived homonegativity in the community among Brazilian gay men, found that more than 93% of participants believe that society punishes homosexual people and 98.55% that discrimination against LGBTQIA+ people is still common.

Brazilian gay men tend to be socialized, from early childhood onwards, and exposed to messages in which homosexuality, in all its different manifestations, is considered inferior and undesirable, or even as a disease or sin. One of the most pervasive consequences of chronic exposure to homonegativity is the internalization of stigma by gay [22]. Internalized homonegativity refers to a process that occurs exclusively in non-heterosexual individuals who internalize negative beliefs and attitudes about homosexuality in relation to themselves, as well as towards other homosexual people and homosexuality more broadly [22]. The internalization of sexual stigma is associated with shame and guilt for thoughts and behaviors associated with homoaffectivity [23], low self-esteem [24], psychological distress [24,25], feelings of inadequacy and loneliness [25], restricted social support network associated with deficits in socio-emotional skills and fear of intimacy [26,27] lower satisfaction in affective–sexual relationships [28], and psychopathologies [25,29]. Part of homonegativity, which is highly widespread in different Brazilian social contexts, is related to religious narratives which associate this form of sexuality, openly or in a pernicious and veiled way, with sin, moral deviance or consequence of mistakes made in previous incarnations [30,31,32,33].

The Brazilian population is markedly religious. According to the latest sociodemographic census, the Brazilian population is predominantly Christian, Catholic, or Evangelical [34]. In a recent study evaluating religiosity and spirituality in 26 countries in the northern and southern hemispheres, it was found that Brazil ranked first in terms of prevalence of religious belief, with 89% of participants believing in a higher being and 76% reporting having a religion [35].

Both the Catholic and Protestant churches have the “Holy Bible” as their dogmatic basis, a book in which homosexuality is portrayed as a deviation, an unnatural manifestation of sexuality, considered a sin, to be punished with social ostracism, violence, and death. Especially in recent decades, messages prevalent in Brazilian religious institutions, such as “God loves sinners, but hates sin”, have been used to justify a stance of apparent acceptance of homosexual individuals within religious institutions [36]. However, this apparent acceptance is normally associated with the disapproval of homosexuality and conditioned by the recommendation of repression and sublimation of sexuality, which can lead to various forms of social and psychosexual distress [37,38,39,40,41,42].

The panorama of the religious experience of sexual minorities in the context of Spiritist religions does not differ substantially from the reality observed in the Christian context in Brazil [32,33,37]. Pereira [33] points out that, while Allan Kardec’s doctrine and the teachings of influential mediums, such as Chico Xavier and Divaldo Franco, offer a neutral view that considers homosexuality, this approach is not unanimous among practitioners of Spiritism. In some groups of this religion, homosexuality is understood as a consequence of morally deviant behaviors in past lives, as a way of “paying a symbolic debt” [43]. African-based religions, such as Candomblé and Umbanda, present, apparently, a more inclusive and less normative approach to sexual diversity [33]. In these traditions, spiritual essence is not related to gender identity or sexual orientation, transcending gender duality. Institutional dogmas, therefore, do not impose rigid norms of sexual conduct [33]. However, in Brazil, religions of African origin are influenced by Catholicism and Spiritualism, which may include the notion of homosexuality as a manifestation of less developed sexuality in spiritual terms. Therefore, no Brazilian religion, among the most predominant in Brazil, adopts an institutionally openly affirmative position in relation to sexual and gender diversity.

In the last few decades, a reduction in the number of Catholic individuals has been observed, as well as the emergence of new ultra-conservative evangelical denominations, a phenomenon more prevalent among low-income communities, coming from the outskirts of large urban centers or small towns in the interior of Brazilian states [35]. In dogmatic terms, there were changes in the partial relaxation of Catholic dogmas in the understanding of homosexuality, and a hardening of evangelical dogmas [36,37].

The influence of religious principles in the daily life of Brazilians goes beyond the confines of churches, communities or congregations and is present in different social contexts [44,45]. These religious dogmas can be seen, for example, in the formation of political parties made up predominantly of evangelical individuals, who defend agendas that hinder progress in the field of human rights for sexual minorities in Brazil, such as the right to marriage and the adoption of children by same-sex couples [45,46].

In a recent integrative literature review, which evaluated the effects of religiosity and spirituality on the perception of internalized homonegativity and mental health indicators in the LGBTQIA+ population, based on the analysis of 27 studies, the authors found contradictory results, which point to religiosity and spirituality as both a risk and protective factors for negative mental health outcomes, including the internalization of homonegativity and the increase in cognitive dissonance between religiosity/spirituality and sexuality [44]. The researchers point out that two variables may explain the controversial results. The first has to do with the dogmatic nature of religion itself, in terms of affirmation or prejudice and marginalization of sexual minorities. The second has to do with how the individual who belongs to a religious denomination relates to dogma and beliefs. The authors argue that inflexible beliefs that are not open to reflection and relativization tend to produce negative outcomes, while private spiritual practices that relativize the rules of conduct tend to produce more favorable outcomes. It is important to note that most of the studies selected in this literature review were carried out in developed, English-speaking countries. None of the 27 articles selected evaluated associations between religion, homonegativity and depression in Brazilian gay men, which highlights a gap in the representativeness of research in the area.

Brazil is a markedly religious country and there is no shortage of evidence of intolerance, violence and marginalization of gay men naturalized by religious narratives that associate homosexuality with immorality and sin. Therefore, investigating associations between the internalization of homonegativity and its possible associations with the religiosity of gay men is a relevant measure, as it produces useful knowledge for mental health care to meet the specific needs of this population. Hence, the aims of our study were as follows: (1) to assess possible differences in perceived and internalized homonegativity in the community and depression reported by gay men who declared themselves Christians, Spiritualists and Atheists or Agnostics; (2) to assess possible associations between homonegativity and depression in the sample as a whole; (3) to assess the predictive and mediative power of religion in homonegativity and depression. We hypothesize that there will be significant differences in terms of homonegativity and depression between the groups, according to religious affiliation, with a greater perception of community and internalized homonegativity in the groups with nominal religion, compared to the group of Atheists and Agnostics. In addition, we believe that we will find positive and significant correlations between homonegativity and depression when evaluating the overall sample, and that religious affiliations will be a significant predictor of homonegativity and depression.

## 2. Materials and Methods

This was a cross-sectional study. Our project was conducted at the School of Nursing, University of São Paulo, Brazil. The sample consisted of 194 self-identified Brazilian gay men, with an average age of 35.5 years (SD = 8.83). This was a non-probabilistic sample, based on convenience criteria. The participants were recruited on social media such as Instagram and Facebook. The inclusion criteria were as follows: being male and having sex with other men, being over 18 years old and having access to the Internet and the possibility of filling out the assessment instruments in privacy. The researcher interviewed participants to evaluate whether they met the inclusion criteria.

### 2.1. Measurement Instruments

Internalized Homophobia Scale: This questionnaire evaluated two dimensions: internal and external perception of stigma [47]. All items were written in affirmative form and measurement was carried out through a five-point Likert scale ranging from 1 (strongly disagree) to 5 (strongly agree). Examples of the statements are as follows: (1) Typically, effeminate gay men make me feel uncomfortable; (2) I prefer to have anonymous sexual partners; (3) Life would be easier if I were heterosexual. Higher scores indicated higher levels of internalized homophobia. No cut-off point exists for the classification of homophobia. In the present study, we used the 19-item Brazilian version of the scale, which showed better internal validity in a validation article [48], with a Cronbach’s alpha of 0.814 for the internal perception of stigma and 0.622 for the external perception of stigma.

Depression Inventory (BDI-II): The instrument comprises 21 items, each with four alternatives [49]. The questions encompass physical symptoms, such as fatigue, sleep, and weight alterations, and cognitive alterations verified in patients diagnosed with depression, such as persistent sadness, pessimism, feelings of failure, dissatisfaction, and guilt. The level of depression was classified according to the total score: 0–11 = minimal, 12–19 = mild, 20–35 = moderate, and 36–63 = severe. In a validation study of the instrument in a Brazilian population, a Cronbach’s alpha of 0.81 was obtained [50].

Sociodemographic Questionnaire: this instrument was developed by the first author, and it assesses the sociodemographic characteristics of participants (age, housing, marital status, education, occupation, and income).

### 2.2. Procedures and Ethical Considerations

The participants completed the instruments online, in the first half of 2023, anonymously. Completing the instruments took approximately 30 min. The participants were selected from Facebook groups aimed at the LGBTQIA+ population, where the research protocol was periodically published over the course of three months. This study was approved by the Research Ethics Committee of the School of Nursing, University of São Paulo (number: 4.601.952, CAAE: 31527820.7.0000.5392; 19 March 2021). All participants provided written informed consent.

### 2.3. Data Analysis

Statistical analyses were performed using SPSS 29.0, and significance was set at 5% (*p* < 0.05). Descriptive analyses were presented as frequencies, proportions, means, medians, and standard deviations. Data normality was assessed using the Shapiro–Wilk tests, and homogeneity of variance was assessed using Levene’s test. Once the data distribution of the 194 participants was classified as normal, correlation analyses were performed between depression and homophobia using Pearson’s r test. The following intervals were adopted to classify the intensity of the correlation between the variables analyzed: from 0 to 0.30, slight correlation; from 0.30 to 0.70, moderate correlation; and from 0.7 to 1, strong correlation between variables [51]. The sample was divided into three groups: Christians, Spiritualists and Atheists/Agnostics. The groups were divided based on the following question: “Do you consider yourself to belong to a religious denomination? If so, which one?” Differences in sociodemographic variables and in terms of homophobia and depression between the three groups were evaluated using the chi-square and ANOVA tests. To evaluate the predictive power of religion on homonegativity and depression, we conducted a linear regression. To determine whether the relationship between internalized homonegativity and depression symptoms was mediated by religious affiliation, we conducted a mediation analysis using PROCESS [52].

## 3. Results

In total, 194 Brazilian gay men recruited through social networks took part in our study. The average age of the participants was 35.67 (SD = 8.83) years. Our participants were mostly single (74.5%) and white (72.2%). The majority had an income between BRL 2000 and BRL 4999, corresponding to EUR 360 and EUR 900 and USD 400 and USD 1000, (36.2%), completed higher education (78.9%), were employed (88.7%) and lived with family members or partners (53.1%), in rented accommodation (50.5%). Table 1 shows the sociodemographic characteristics of the general sample and those divided into the groups of individuals who declared themselves Christians (n = 71), with an average age of 35.13 years (SD = 9.04), Spiritualists (n = 52), with an average age of 38.96 years (SD = 8.82), and Atheists or Agnostics (n = 71), with an average age of 33.89 (SD = 8.09).

We carried out Levene’s test to assess the homogeneity between the three groups, formed on the basis of self-declared religious affiliation. The group proved to be homogeneous, with differences only in the variables of marital status, with a higher number of married Christians, and skin color, with a higher frequency of white individuals in the group of Atheists and Agnostics. We found high scores of perceived homonegativity in the community and internalized homonegativity in the sample. We identified higher scores of perceived social homonegativity in the Atheist/Agnostic group, but this difference did not reach statistical significance. Higher scores of internalized homonegativity and total homonegativity were identified in the group of Christians and Spiritualists, with statistically significant differences (Table 2).

More than 60% of the overall sample showed some level of depression. The majority of participants who did not show signs and symptoms of depression were Atheists or Agnostics. Christians were more likely to show mild and moderate depression, and severe depression was more common among Spiritualists. No statistically significant differences were identified in terms of the proportion of participants with mild, moderate or severe depression according to religious affiliation. Table 3 shows the correlations between depression scores and homonegativity in the total sample.

We found a positive, moderate and statistically significant correlation between depression and total and internalized homonegativity, as well as between depression and social homonegativity, although the latter correlation was weaker. To assess the contribution of sociodemographic variables (age, education, skin color and marital status), social homonegativity and internalized homonegativity, and religion to symptoms of depression, a three-model hierarchical linear regression was conducted. Looking at the third model, it was found that the main predictors of the occurrence of symptoms of depression were education (β = −0.181; *p* = < 0.05) and internalized homonegativity (β = 0.313; *p* = < 0.001). These results can be seen in more detail in Table 4.

**Figure 1:**
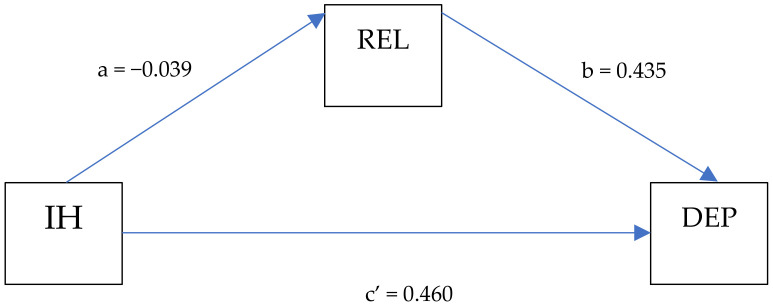
Simple mediation model. Source: own elaboration.

To determine whether the relationship between internalized homonegativity and symptoms of depression was mediated by religious affiliation, we conducted a mediation analysis using PROCESS [53] Regarding the direct effect of internalized homonegativity on symptoms of depression, internalized homonegativity was found to be a significant predictor of symptoms of depression (β = 0.460; *p* = < 0.001) (Figure 1). 

With the inclusion of the mediator (religious affiliation), the indirect effect of internalized homonegativity on symptoms of depression is not statistically different from zero, as shown by the confidence interval that includes zero [ab = (−0.039 × 0.435) = −0.017], meaning that religious affiliation does not mediate the relationship between internalized homonegativity and symptoms of depression (Table 5).

## 4. Discussion

Our study aimed to evaluate indicators of perceived internalized homophobia in the community and signs/symptoms of depression reported by Brazilian gay men with a nominal religion and compare them to those reported by Atheists or Agnostics. In addition, we evaluated possible correlations between homonegativity and depression in the sample as a whole and the predictive power of membership of the Christian and Spiritism religions, the most frequent in Brazil, for perceived, internalized homonegativity and depression.

We found elevated depression scores in the group of gay men both with and without religion filiation. Although we found higher depression scores among gay men with a religious connection compared to Agnostic and Atheist participants, this difference was not significant. This result contradicts our initial hypothesis, and we consider it may be due to the influence of other social and cultural factors related to sexual stigma, which affect all groups equally, regardless of religion. We also found significant differences between total and internalized homonegativity scores between groups, with gay Brazilian men who declared affiliation to Christian or Spiritist religions showing higher indicators of homonegativity. This result corroborates previous studies on the negative, pervasive and long-lasting effects of the attachment of sexual and gender minorities to a religious institution with non-affirmative dogmas [53,54].

In Brazil, Spiritist and Christian religious narratives continue to be marked by a prejudiced and homonegative discourse, more explicit in Christian denominations, such as Catholicism and Protestantism, which associate homosexuality with sin, immorality, promiscuity, unhappiness, and loneliness [31] and less explicit in Spiritualist denominations, which usually associate homosexuality as the “price one pays” in the current incarnation for mistakes made in previous incarnations [33]. We believe that the effects of the homonegative religious narrative are especially relevant in Brazil because the country is among those with the highest number of people who report having a formal religious affiliation and private spiritual practices [35], and most of these individuals are affiliated with Christian religions—Catholic and Protestant—with prejudiced narratives about homo-affectivity [35,36].

Although the Catholic Church has, in recent years, mainly due to the influence of Pope Francisco, adopted an apparently more progressive stance towards sexual and gender diversity, the messages are still inconsistent. For example, in one interview, the Pope says that homosexuals must be respected and included in the Catholic Church, and in another, he refuses the possibility of blessing unions between people of the same sex by the Catholic Church. This discrepancy between the leaders’ discourse and institutional practices, associated with the reception and acceptance of homosexual individuals conditioned to the suppression of the individual’s sexual behavior and sublimation of sexual life, implies psychosexual impairment and emotional distress. It also favors the feeling of inadequacy, guilt, shame related to homosexuality, the internalization of homonegativity and the emergence or worsening of mental disorders among gay individuals [38,39,40,41].

Furthermore, it is important to highlight that the stance of tolerance and conditioned acceptance found in both Christian and spiritualist religions in Brazil is quite different from an affirmative stance in relation to sexual diversity, which is evidenced in the contradictory messages and in the implicit bias in statements by religious leaders. We believe that this contradictory message pattern favors cognitive dissonance among gay men, and hinders the perception of internalized homonegativity associated with belonging to religions that do not affirm sexual diversity.

The effects of homonegativity on religion in Brazil are expressed beyond the context of immediate social relations, and have been verified in recent years by the emergence of far-right religious groups, which have organized themselves into political parties whose agendas include reducing the visibility of LGBTQIA+ movements [31,45] as well as suppressing rights acquired in recent decades [17,19,20,46]. We believe that both the homonegative messages propagated by these religious movements and the formation of political parties with homophobic narratives, in addition to producing various mental health damages in gay individuals, as described above, naturalize and justify violence against sexual and gender minorities, and encourage the population to engage in homophobic behavior.

To deepen our understanding of the relationships between homonegativity, depression and sociodemographic variables, we carried out a three-model hierarchical linear regression, the results of which showed that the main predictors of depression symptoms were education and homonegativity. These findings reinforce the idea that education can act as a protective factor against depression, while homonegativity represents a significant risk factor. However, our results did not indicate significant differences between religion and depression in the groups, as although, descriptively, depression scores were lower among Atheist and Agnostic participants compared to those reported by Christians and Spiritualists, this difference did not reach statistical significance. These data are corroborated by our mediation analysis which showed that homonegativity is a significant predictor of depression symptoms, but religious affiliation did not mediate this relationship. This indicates that the impact of homonegativity on depressive symptoms is direct and not influenced by religious affiliation. This result contradicts previous studies, which indicate that sexual minorities in non-affirmative religions tend to present signs and symptoms of depression more frequently, as well as other manifestations of psychological distress [53,55].

Moreover, in a large study [56], which evaluated more than 21,000 young American adults, the authors found that gay and lesbian participants who considered a non-affirmative religion to be very important had more suicidal ideation and suicide attempts throughout their lives compared to heterosexual individuals. The authors therefore concluded that the importance attributed to non-affirmative religious practice, in addition to not preventing suicide among gays and lesbians, is a risk factor for this outcome among gays and lesbians. In view of the above, we believe that our results regarding the absence of a statistically significant difference between groups with and without religious affiliation should be analyzed with caution because we believe that this is due both to the relatively small size of our sample and to the possibility that depression is modulated by several other variables that we did not control for in this study, such as social support, access to mental health services such as psychotherapy and drug treatment, and aspects related to the religious and spiritual experience of individuals.

Corroborating our second hypothesis, we found positive and significant correlations between homonegativity and depression in the general sample. This result is reiterated by previous studies, which indicate that the homonegativity to which gay individuals are exposed from the first years of their socialization, expressed through physical, verbal and symbolic violence, as well as through microaggressions aimed at the gay individual themselves or other members of the community, is associated with a negative self-concept, low self-esteem, loneliness, social isolation, emotional distress, substance abuse disorder, and post-traumatic stress disorder, among other mental disorders [8,9,20,57,58]. In addition, chronic exposure to homonegativity increases the likelihood of internalization of stigma by gay men, a phenomenon associated with various negative outcomes in terms of physical and mental health, such as shame or guilt for thoughts and behaviors associated with homoaffectivity, frequency of risky sexual behaviors, poor self-care, non-adherence to behavioral and drug treatments, social isolation associated with the expectation of rejection, stress caused by the need to hide sexual orientation, and non-disclosure of sexual orientation even in more welcoming or affirmative environments [8,9,20,57,58].

In the literature review carried out by Rosa and Esperândio [44], contradictory results were evident regarding the relationship between religiosity and spirituality and the mental health of sexual minorities. We believe that the results on the influence of religious affiliation on depression and homonegativity have been contradictory because these outcomes may in fact be different and are probably modulated by several variables. For example, a gay man’s attachment to a religious entity and the practice of spirituality can produce mental health gains associated with positive coping, related to belief and trust in a higher being, a sense of belonging and protection. Particularly if the representatives of this religion have a more affirmative stance on sexual and gender diversity, these benefits are more likely. However, negative outcomes in terms of low self-esteem, a sense of inadequacy and homonegativity are more likely if the religious affiliation emphasizes a superior being who watches over, judges and punishes according to static and rigid precepts that do not value diversity, and there is therefore a probable association between the style of attachment to religion and its superior and the mental health outcomes of sexual minorities [44,53,55,56].

Additionally, the influence of religious affiliation on the mental health of minorities also depends on how the individual relates to their own experience within a religious belief; for example, a rigid, dogmatic and inflexible commitment to a religion that does not affirm diversity is likely to produce deleterious effects in terms of depression and homonegativity, while a commitment that relativizes and selectively incorporates the precepts that make sense, with some flexibility, may be associated with non-deleterious or even favorable outcomes, although there is a risk of cognitive dissonance between religious practice and the experience of homoaffectivity. However, positive coping among sexual minorities who are part of religions that do not affirm sexual diversity tends to be much less likely [44].

Furthermore, several studies have demonstrated the possibility that gay men who adhere to a religion that does not affirm sexual diversity find ways to accommodate the dissonance between their religiosity and sexuality [23,44]. Among these forms of accommodation and coping with the conflict between religiosity and sexuality, researchers point out the selective rejection of non-affirmative aspects of the religious tradition of choice, and the search for more progressive religious centers. These institutions tend to be less focused on the notions of sin, hell, and more guided by the notion of a welcoming and loving God, and not an authoritarian one, and include practical messages about quality of life and spiritual comfort.

To the best of our knowledge, our study is one of the first to evaluate associations between religiosity, homonegativity and depression among Brazilian gay men. Although we believe that our objectives have been achieved, we must acknowledge this study’s limitations. Our study has a cross-sectional design, which precludes establishing causal relationships between religious affiliation, depression and homonegativity. Our sample was selected based on convenience criteria, and the participants are mostly white, and of relatively high socioeconomic status. This profile of participants does not represent the population of Brazilian gay men, and, therefore, generalizations should be made with caution. Our sample was relatively small, which may be related to the fact that we did not find significant differences between groups in levels of depression.

Furthermore, we used self-report instruments that were used to measure homonegativity and depression, which may have implied a risk of being underestimated by the participants’ capacity for self-perception. To divide the groups of Atheists/Agnostics, Christians, and Spiritualists, we used only one question about religiosity. We did not evaluate important aspects in this study, such as the level of engagement in institutional religious practices or level of adherence to private spiritual practices. We also did not evaluate variables that could, at least partially, explain beneficial or negative effects related to having a religious affiliation, nor did we evaluate how everyone relates to the dogmas of each religion, which can vary widely in terms of strict adherence, inflexibility, and dogmatism, to the relativization of beliefs and practices, as well as flexibility and some level of criticism of beliefs based on of the individual’s own criteria. Finally, we group both Catholic and Protestant individuals under the generic designation of “Christians”, which carries the risk of inappropriate generalizations, given that these religious denominations differ significantly in dogmatic terms. Furthermore, it is important to consider the possible sociocultural and demographic influences of different regions of Brazil, a country of continental dimensions, characterized by profound regional heterogeneity and one of the highest rates of social inequality in the world. Therefore, the inclusion of Catholics and Protestants in a single group can lead to conclusions that do not fully reflect the reality experienced by these individuals. Dogmatic differences, combined with regional specificities and demographic variables, suggest that a more segmented analysis would be necessary to capture the diverse influences that shape religiosity and attitudes towards sexual diversity in Brazil. This would avoid simplifications and allow for a deeper and more accurate understanding of the social and cultural dynamics in question.

Hence, conducting replication studies with larger samples from all regions of Brazil and longitudinal research will be crucial to deepening our understanding of the relationship between religiosity, stigma, and mental health among Brazilian gay men. Finally, studies that investigate variables such as the level of flexibility in incorporating beliefs and dogmas, level of engagement in institutional religious activities and private spiritual practices, both in qualitative and quantitative studies, are necessary. For future studies, we also consider necessary those that evaluate the specificities of the beliefs, feelings, and behaviors of Catholic and Protestant Christians regarding homosexuality, as well as the impact of these positions on Brazilian gay men who practice these religions. A detailed understanding of the differences between these religious denominations is important to identify the different ways in which their doctrines and practices influence the experience of sexuality among their followers. The same reasoning applies to the analysis of the various spiritual and African-based religious entities. These traditions, which have beliefs and practices distinct from Christian denominations, also present varied approaches to homosexuality, reflecting the cultural and spiritual diversity of Brazil. Quantitative and qualitative studies that explore these differences are essential for a more comprehensive understanding of the effects of religions on the formation of sexual identity and the mental health of gay individuals.

## 5. Conclusions

Our study responds to a call for investigation of variables that contribute to mental health inequalities among a population of minorities. We evaluated a sample of Brazilian gay men and identified interesting associations between religious affiliation, perceived community and internalized homonegativity, and depression. Especially among minorities from underdeveloped or developing countries, these studies are scarce. Our data indicated a high prevalence of depression in the sample of Brazilian gay men. Regardless of religious affiliation, more than 60% of participants showed signs and symptoms of depression at a clinical level. We also found higher indicators of internalized homonegativity among gay men who declared themselves Christians or Spiritualists, religious institutions that present, among their dogmas, a negative or controversial understanding regarding homoaffectivity in its various forms of manifestation. Therefore, our results suggest that gay men’s chronic exposure to non-affirming religious affiliation contexts may harm the construction of a positive gay identity and should be taken into consideration when addressing the mental health inequalities of sexual minorities.

## Figures and Tables

**Table 1:** Sociodemographic description of participants according to self-declared religious denomination.

		Total (n = 194)		Christians (n = 71)		Spiritualists (n = 52)		Atheists/ Agnostics (n = 71)	
		N	%	n	%	n	%	n	%
Marital status *	Single	143	74.5	55	77.5	41	78.8	47	68.1
	Married	43	22.4	13	18.3	11	21.2	19	27.5
	Divorced	6	3.1	3	4.2	0	0	3	4.3
Skin color	White	140	72.2	47	66.2	37	71.2	56	78.9
	Black or Brown	54	27.8	24	33.8	15	28.8	15	21.1
Education	Elementary	1	0.5	1	1.4	0	0	0	0
	Middle/Secondary	16	18.6	16	22.5	5	9.6	15	21.1
	Technical	33	2.1	1	1.4	2	3.8	1	1.4
	College	32	33	24	33.8	16	30.8	24	33.8
	Postgraduate	25	25.3	18	25.4	16	30.8	15	21.1
	Master	2	10.8	6	8.5	6	11.5	9	12.7
	Doctorate	4	9.8	5	7	7	13.5	7	9.9
Housing	Rented	98	50.5	37	52.1	23	44.2	38	53.5
	Owned	84	43.3	32	45.1	24	46.2	28	39.4
	Borrowed/Lent	12	6.2	2	2.8	5	9.6	5	7
Cohabitation	Alone	70	36.1	29	40.8	22	42.3	19	26.8
	family/partner	103	53.1	35	49.3	24	46.2	44	62
	With colleagues/friends	21	10.8	7	9.9	6	11.5	8	11.3
Socio-economic status **	BRL 1300–1999 (EUR 234–EUR 360; USD 260–USD 400)	12	6.8	7	10.3	1	2	4	6.8
	BRL 2000–4999 (EUR 360–EUR 900; USD 400–USD 1000)	64	36.2	28	41.2	14	28	22	37.3
	BRL 5000–9999 (EUR 900–EUR 1.800; USD 1000–USD 2000)	44	24.9	11	16.2	16	32	17	28.8
	BRL 10,000–14,999 (EUR 1800–EUR 2700; USD 2000–USD 3000)	18	10.2	9	13.2	5	10	4	6.8
	>BRL 15,000 (>EUR 2700; >USD 3000)	39	22	13	19.1	14	28	12	20.3
Professional situation	Unemployed	20	10.3	6	8.5	7	13.5	7	9.9
	Employed	172	88.7	64	90.1	45	86.5	63	88.7
	Retired	2	1	1	1.4	0	0	1	1.4

Source: own elaboration. * Marital status assessed in 192 participants; ** Socioeconomic status assessed in 177 participants. We excluded 10 participants with incomes below the national minimum wage in 2023. Two participants who did not report their income and four outlier participants reported incomes above BRL 40,000. Values converted using the exchange rate of 22 May 2024.

**Table 2:** Mean depression homonegativity scores and between groups according to religious affiliation.

	Total Sample (n = 194)	Christians (n = 71)	Spiritualists (n = 52)	Atheists/ Agnostics (n = 71)	F(df)	*p*
Depression	15.69 (9.36)	15.9 (8.46)	16.79 (9.93)	14.66 (9.81)	0.802 (2; 191)	0.450
Total homonegativity	43.58 (6.92)	45.77 (6.57)	43.17 (7.69)	41.68 (6.11)	6.723 (2; 191)	0.002 *
Internalized homonegativity	30.71 (6.44)	33.06 (6.13)	30.35 (6.93)	28.63 (5.63)	9.212 (2; 191)	0.000 **
Social homonegativity	12.87 (1.83)	12.72 (1.77)	12.83 (1.84)	13.04 (1.87)	0.570 (2; 191)	0.567

Source: own elaboration. * *p* < 0.05 ** *p* < 0.001.

**Table 3:** Correlations between depression, total homonegativity, internalized homonegativity and social homonegativity.

Variables	1	2	3	4
1. Depression	-			
2. Total homonegativity	0.339 **	-		
3. Internalized Homonegativity	0.316 **	0.965 **	-	
4. Social Homonegativity	0.170 *	0.387 **	0.131	-

Source: own elaboration. * *p* < 0.05 ** *p* < 0.001.

**Table 4:** Hierarchical linear regression models.

	Model 1			Model 2			Model 3		
	B	SE B	β	B	SE B	β	B	SE B	β
Age	−0.008	0.081	−0.007	0.006	0.078	0.006	0.009	0.078	0.009
Education	−1.000	0.465	−0.160 *	−1.111	0.443	−0.178 *	−1.135	0.446	−0.181 *
Skin color	1.480	1.511	0.071	0.593	1.448	0.028	0.664	1.457	0.032
Marital Status	−2.714	1.335	−0.151 *	−1.826	1.282	−0.101	−1.850	1.286	−0.103
Social Homophobia				0.433	0.355	0.085	0.414	0.358	0.081
Internalized Homophobia				0.436	0.101	0.302 **	0.452	0.106	0.313 **
Religion							0.396	0.787	0.036
R^2^	0.065			0.165			0.166		
F for change in R^2^	3.175 *			5.994 **			5.153 **		

Source: own elaboration. * *p* < 0.05 ** *p* < 0.001.

**Table 5:** Total and indirect effects.

Effect	Path	B	SE	BootLLCI	BootULCI	t	*p*
Total	IH -> DEP	0.460	0.099	0.263	0.656	4.616	<0.001
Indirect	IH -> REL -> DEP	−0.017	0.310	−0.082	0.044		

## Data Availability

Data will be available upon request.
